# 3-(3-Meth­oxy­benzyl­idene)chroman-4-one

**DOI:** 10.1107/S160053681200949X

**Published:** 2012-03-10

**Authors:** Kaalin Gopaul, Mahidansha Shaikh, Deresh Ramjugernath, Neil A. Koorbanally, Bernard Omondi

**Affiliations:** a School of Chemistry and Physics, University of KwaZulu-Natal, Westville Campus, Private Bag X54001, Durban 4000, South Africa; b School of Engineering, University of KwaZulu-Natal, Howard College Campus, Private Bag X54001, Durban 4000, South Africa

## Abstract

In the title compound, C_17_H_14_O_3_, the dihedral angle between the meth­oxy­benzene unit and the benzene ring of the chromanone system is 64.12 (3)°. The crystal structure is stabilized by weak C—H⋯O inter­actions.

## Related literature

For the preparation, see: Shaikh *et al.* (2011 ▶). For related structures, see: Kirkiacharian *et al.* (1984 ▶); Marx *et al.* (2008 ▶); Suresh *et al.* (2007 ▶); Chantrapromma *et al.* (2006 ▶); Augustine *et al.* (2008 ▶). For the biological activity of this class of compound, see: du Toit *et al.* (2010 ▶).
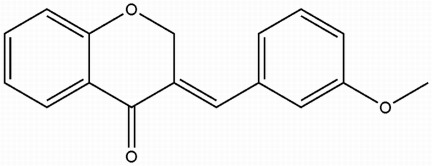


## Experimental

### 

#### Crystal data

C_17_H_14_O_3_
*M* *_r_* = 266.28
Monoclinic, 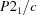

*a* = 12.4143 (9) Å
*b* = 6.7141 (5) Å
*c* = 16.0031 (10) Å
β = 98.658 (4)°
*V* = 1318.67 (16) Å^3^
*Z* = 4
Mo *K*α radiation
μ = 0.09 mm^−1^
*T* = 446 K
0.28 × 0.21 × 0.05 mm

#### Data collection

Nonius KappaCCD diffractometer
4414 measured reflections
2315 independent reflections
1662 reflections with *I* > 2σ(*I*)
*R*_int_ = 0.023

#### Refinement

*R*[*F* ^2^ > 2σ(*F* ^2^)] = 0.041
*wR*(*F* ^2^) = 0.112
*S* = 1.00
2315 reflections
182 parameters
H-atom parameters constrained
Δρ_max_ = 0.21 e Å^−3^
Δρ_min_ = −0.20 e Å^−3^

### 

Data collection: *COLLECT* (Nonius, 1998 ▶); cell refinement: *DENZO-SMN* (Otwinowski & Minor, 1997 ▶); data reduction: *DENZO-SMN*; program(s) used to solve structure: *SHELXS97* (Sheldrick, 2008 ▶); program(s) used to refine structure: *SHELXL97* (Sheldrick, 2008 ▶); molecular graphics: *ORTEP-3* (Farrugia, 1997 ▶); software used to prepare material for publication: *WinGX* (Farrugia, 1999 ▶).

## Supplementary Material

Crystal structure: contains datablock(s) global, I. DOI: 10.1107/S160053681200949X/fj2528sup1.cif

Structure factors: contains datablock(s) I. DOI: 10.1107/S160053681200949X/fj2528Isup2.hkl

Supplementary material file. DOI: 10.1107/S160053681200949X/fj2528Isup3.cml

Additional supplementary materials:  crystallographic information; 3D view; checkCIF report

## Figures and Tables

**Table 1:** Hydrogen-bond geometry (Å, °)

*D*—H⋯*A*	*D*—H	H⋯*A*	*D*⋯*A*	*D*—H⋯*A*
C2—H2*B*⋯O1^i^	0.97	2.54	3.3808 (19)	145
C18—H18*B*⋯O3^ii^	0.96	2.50	3.4227 (19)	161

Symmetry codes: (i) 
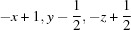
; (ii) 
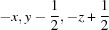
.

**Fig. 1.**
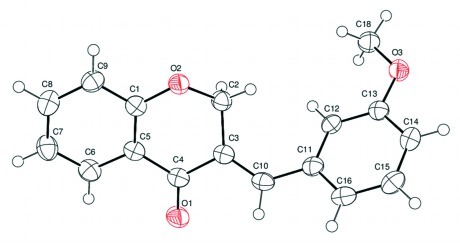
The asymmetric unit of the title compound with displacement ellipsoids drawn at the 50% probability level.

## supplementary crystallographic information

### Comment

The title compound, 3-(3-Methoxybenzylidene)-chroman-4-one, belongs to a class of compounds called homoisoflavonoids, which are C-16, α,β unsaturated carbonyl compounds containing two aromatic rings. They are a group of naturally occurring molecules that are structurally related to isoflavonoids but differ by containing one more carbon atom (Kirkiacharian *et al.*, 1984). Homoisoflavonoids may be categorized into four groups depending on the type of structural backbone present. The four groups are 3-benzylidene-4-chromanones, of which the title compound belongs to as well as the 3-benzyl-4-chromanones, 3-benzyl-3-hydroxy-4-chromanones and scillascillins (du Toit *et al.*, 2010).

This compound may undergo chemical conversion into the (E)- and (*Z*)-isomers (Kirkiacharian *et al.*, 1984). The 3-benzylidene-4-chromanones have been shown to display a wide range of biological activities (du Toit *et al.*, 2010). The most commonly used procedure for the synthesis of homoisoflavoinoids involves the condensation of chroman-4-one with an aromatic aldehyde in the presence of an acidic or basic catalyst (Shaikh *et al.*, 2011).

In the molecular structure, the dihedral angle between the methoxybenzene moeity and the benzene ring of the chromanone moiety is 64.12 (3) °.The Chromanone moiety is fused with a phenyl ring and adopts a half chair conformation (Fig 1). The molecule of (I) is stablized by two weak C—H···O intramolecular interactions (Table 1).

### Experimental

A mixture of chroman-4-one (1 g, 6.749 mmol), 3-methoxybenzaldehyde (1.103 g, 8.099 mmol) and 10–15 drops of piperidine was heated at 80°C for 20 hrs. The reaction mixture was monitored for completion by thin layer chromatography. Upon completion, the reaction mixture was cooled, diluted with water and neutralized using 10% HCl. The reaction mixture was extracted with ethyl acetate (3 × 30 mL). The ethyl acetate layers were combined, washed with brine (20 ml), water (2 × 10 mL) and dried over anhydrous magnesium sulfate. The solvent was reduced and the compound purified by column chromatography using silica gel (Merck 9385, 40–63 µm particle size) with a mobile phase of 2% ethyl acetate in hexane to yield the title compound with a m.p. of 85–86°C.

^1^H NMR: δ (ppm): 3.83 (3*H*, s, OCH_3_), 5.36 (2*H*, d, *J* = 1.72 Hz, 2*H*-2), 6.82 (1*H*, s, H-2'), 6.87 (1*H*, d, *J* = 7.60 Hz, H-6'), 6.93 (2*H*, m, H-8, H-4'), 7.05 (1*H*, t, *J* = 7.52 Hz, H-6), 7.34 (1*H*, t, *J* = 7.92 Hz, H-5'), 7.47 (1*H*, t, *J* = 8.52 Hz, H-7), 7.82 (1*H*, s, H-9), 8.00 (1*H*, dd, *J* = 7.82, 1.46 Hz, H-5). ^13^C NMR: δ (ppm): 55.36 (OCH_3_), 67.66 (C-2), 115.06 (C-4'), 115.42 (C-2'), 117.93 (C-8), 121.92 (C-6), 122.02 (C-4a), 122.28 (C-6'), 127.96 (C-5), 129.76 (C-5'), 131.15 (C-3), 135.69 (C-1'), 135.90 (C-7), 137.40 (C-9), 159.69 (C-3'), 161.18 (C-8a), 182.23(C-4).

### Crystal data

**Table:**

C_17_H_14_O_3_	*F*(000) = 560
*M**_r_* = 266.28	*D*_x_ = 1.341 Mg m^−^^3^
Monoclinic, *P*2_1_/*c*	Mo *K*α radiation, λ = 0.71073 Å
Hall symbol: -P 2ybc	Cell parameters from 5379 reflections
*a* = 12.4143 (9) Å	θ = 2.6–25°
*b* = 6.7141 (5) Å	µ = 0.09 mm^−^^1^
*c* = 16.0031 (10) Å	*T* = 446 K
β = 98.658 (4)°	Block, colourless
*V* = 1318.67 (16) Å^3^	0.28 × 0.21 × 0.05 mm
*Z* = 4	

### Data collection

**Table:**

Nonius KappaCCD diffractometer	*R*_int_ = 0.023
Graphite monochromator	θ_max_ = 25°, θ_min_ = 2.6°
φ and ω scans	*h* = −14→14
4414 measured reflections	*k* = −7→7
2315 independent reflections	*l* = −19→18
1662 reflections with *I* > 2σ(*I*)	

### Refinement

**Table:**

Refinement on *F*^2^	Primary atom site location: structure-invariant direct methods
Least-squares matrix: full	Secondary atom site location: difference Fourier map
*R*[*F*^2^ > 2σ(*F*^2^)] = 0.041	Hydrogen site location: inferred from neighbouring sites
*wR*(*F*^2^) = 0.112	H-atom parameters constrained
*S* = 1.00	*w* = 1/[σ^2^(*F*_o_^2^) + (0.0753*P*)^2^] where *P* = (*F*_o_^2^ + 2*F*_c_^2^)/3
2315 reflections	(Δ/σ)_max_ < 0.001
182 parameters	Δρ_max_ = 0.21 e Å^−^^3^
0 restraints	Δρ_min_ = −0.20 e Å^−^^3^

### Special details

**Table:**

Experimental. Carbon-bound H-atoms were placed in calculated positions [C—H = 0.96 Å for Me H atoms, 0.97 Å for Methylene H atoms and 0.93 Å for aromatic H atoms; *U*_iso_(H) = 1.2*U*_eq_(C) (1.5 for Me groups)] and were included in the refinement in the riding model approximation.
Geometry. All e.s.d.'s (except the e.s.d. in the dihedral angle between two l.s. planes) are estimated using the full covariance matrix. The cell e.s.d.'s are taken into account individually in the estimation of e.s.d.'s in distances, angles and torsion angles; correlations between e.s.d.'s in cell parameters are only used when they are defined by crystal symmetry. An approximate (isotropic) treatment of cell e.s.d.'s is used for estimating e.s.d.'s involving l.s. planes.
Refinement. Refinement of *F*^2^ against ALL reflections. The weighted *R*-factor *wR* and goodness of fit *S* are based on *F*^2^, conventional *R*-factors *R* are based on *F*, with *F* set to zero for negative *F*^2^. The threshold expression of *F*^2^ > σ(*F*^2^) is used only for calculating *R*-factors(gt) *etc*. and is not relevant to the choice of reflections for refinement. *R*-factors based on *F*^2^ are statistically about twice as large as those based on *F*, and *R*- factors based on ALL data will be even larger. The following ALERTS were generated. Each ALERT has the format test-name_ALERT_alert-type_alert-level. PLAT910_ALERT_3_C Missing # of FCF Reflections Below Th(Min) ···.. 1 PLAT911_ALERT_3_C Missing # FCF Refl Between THmin & STh/*L*= 0.595 3 PLAT913_ALERT_3_C Missing # of Very Strong Reflections in FCF ···. 3 PLAT909_ALERT_3_G Percentage of Observed Data at Theta(Max) still 46 Perc. Noted

### Fractional atomic coordinates and isotropic or equivalent isotropic displacement parameters (Å^2^)

**Table:**

	*x*	*y*	*z*	*U*_iso_*/*U*_eq_	
C1	0.67104 (13)	−0.0244 (2)	0.41235 (9)	0.0308 (4)	
C2	0.48754 (13)	0.0872 (2)	0.36955 (9)	0.0345 (4)	
H2A	0.4209	0.0984	0.3941	0.041*	
H2B	0.47	0.0197	0.3156	0.041*	
C3	0.52909 (12)	0.2927 (2)	0.35484 (8)	0.0311 (4)	
C4	0.64423 (13)	0.3033 (2)	0.33980 (9)	0.0334 (4)	
C5	0.71355 (12)	0.1319 (2)	0.36945 (9)	0.0309 (4)	
C6	0.82476 (13)	0.1305 (2)	0.36328 (9)	0.0384 (4)	
H6	0.8537	0.2318	0.3338	0.046*	
C7	0.89190 (15)	−0.0181 (2)	0.40005 (10)	0.0431 (4)	
H7	0.9659	−0.0166	0.3963	0.052*	
C8	0.84792 (14)	−0.1708 (2)	0.44296 (10)	0.0425 (4)	
H8	0.8933	−0.2712	0.4681	0.051*	
C9	0.73865 (13)	−0.1761 (2)	0.44890 (9)	0.0368 (4)	
H9	0.7102	−0.2801	0.4771	0.044*	
C10	0.47198 (13)	0.4614 (2)	0.35582 (8)	0.0332 (4)	
H10	0.5096	0.578	0.3479	0.04*	
C11	0.35772 (13)	0.4872 (2)	0.36769 (9)	0.0319 (4)	
C12	0.27627 (12)	0.3537 (2)	0.33279 (8)	0.0302 (4)	
H12	0.2937	0.2466	0.3005	0.036*	
C13	0.16990 (12)	0.3818 (2)	0.34652 (8)	0.0307 (4)	
C14	0.14246 (14)	0.5448 (2)	0.39277 (9)	0.0360 (4)	
H14	0.0707	0.5638	0.4012	0.043*	
C15	0.22209 (14)	0.6782 (2)	0.42606 (9)	0.0387 (4)	
H15	0.2038	0.787	0.457	0.046*	
C16	0.32907 (14)	0.6509 (2)	0.41361 (9)	0.0364 (4)	
H16	0.3822	0.7419	0.4359	0.044*	
C18	0.11018 (14)	0.0863 (2)	0.27058 (10)	0.0392 (4)	
H18A	0.1373	0.1281	0.2203	0.059*	
H18B	0.0455	0.008	0.2554	0.059*	
H18C	0.1645	0.0078	0.3048	0.059*	
O1	0.68030 (9)	0.45005 (16)	0.30738 (7)	0.0461 (3)	
O2	0.56502 (9)	−0.03170 (14)	0.42472 (6)	0.0357 (3)	
O3	0.08530 (8)	0.25717 (14)	0.31708 (6)	0.0377 (3)	

### Atomic displacement parameters (Å^2^)

**Table:**

	*U*^11^	*U*^22^	*U*^33^	*U*^12^	*U*^13^	*U*^23^
C1	0.0339 (10)	0.0333 (8)	0.0255 (7)	−0.0017 (7)	0.0053 (7)	−0.0052 (6)
C2	0.0318 (9)	0.0358 (9)	0.0349 (8)	−0.0019 (7)	0.0023 (7)	0.0042 (7)
C3	0.0340 (10)	0.0331 (8)	0.0250 (7)	−0.0038 (7)	0.0010 (7)	0.0028 (6)
C4	0.0364 (10)	0.0350 (8)	0.0284 (8)	−0.0060 (7)	0.0033 (7)	0.0012 (7)
C5	0.0338 (10)	0.0342 (8)	0.0246 (7)	−0.0045 (7)	0.0043 (6)	−0.0018 (6)
C6	0.0393 (11)	0.0442 (9)	0.0328 (8)	−0.0033 (8)	0.0095 (7)	0.0036 (7)
C7	0.0347 (10)	0.0541 (10)	0.0421 (9)	0.0034 (8)	0.0115 (8)	0.0024 (8)
C8	0.0436 (12)	0.0450 (10)	0.0395 (9)	0.0096 (8)	0.0082 (8)	0.0036 (7)
C9	0.0440 (11)	0.0340 (8)	0.0331 (8)	0.0016 (8)	0.0086 (7)	0.0027 (7)
C10	0.0371 (10)	0.0318 (8)	0.0295 (8)	−0.0079 (7)	0.0014 (7)	0.0039 (6)
C11	0.0369 (10)	0.0295 (8)	0.0285 (7)	−0.0001 (7)	0.0031 (7)	0.0063 (6)
C12	0.0357 (10)	0.0273 (8)	0.0272 (7)	0.0025 (7)	0.0033 (7)	0.0006 (6)
C13	0.0327 (10)	0.0304 (8)	0.0279 (7)	−0.0003 (7)	0.0012 (7)	0.0029 (7)
C14	0.0376 (10)	0.0369 (9)	0.0337 (8)	0.0075 (7)	0.0058 (7)	−0.0005 (7)
C15	0.0505 (12)	0.0320 (8)	0.0328 (8)	0.0067 (8)	0.0036 (8)	−0.0029 (7)
C16	0.0459 (11)	0.0281 (8)	0.0331 (8)	−0.0049 (7)	−0.0006 (7)	0.0016 (7)
C18	0.0399 (10)	0.0356 (9)	0.0414 (9)	−0.0022 (7)	0.0035 (8)	−0.0060 (7)
O1	0.0398 (7)	0.0450 (7)	0.0544 (7)	−0.0050 (5)	0.0104 (6)	0.0163 (6)
O2	0.0322 (7)	0.0353 (6)	0.0399 (6)	−0.0008 (5)	0.0064 (5)	0.0092 (5)
O3	0.0320 (7)	0.0366 (6)	0.0442 (6)	0.0002 (5)	0.0051 (5)	−0.0072 (5)

### Geometric parameters (Å, º)

**Table:**

C1—O2	1.3610 (18)	C9—H9	0.93
C1—C9	1.391 (2)	C10—C11	1.469 (2)
C1—C5	1.400 (2)	C10—H10	0.93
C2—O2	1.4443 (18)	C11—C16	1.397 (2)
C2—C3	1.504 (2)	C11—C12	1.403 (2)
C2—H2A	0.97	C12—C13	1.384 (2)
C2—H2B	0.97	C12—H12	0.93
C3—C10	1.337 (2)	C13—O3	1.3697 (18)
C3—C4	1.487 (2)	C13—C14	1.392 (2)
C4—O1	1.2290 (16)	C14—C15	1.380 (2)
C4—C5	1.472 (2)	C14—H14	0.93
C5—C6	1.399 (2)	C15—C16	1.384 (2)
C6—C7	1.375 (2)	C15—H15	0.93
C6—H6	0.93	C16—H16	0.93
C7—C8	1.391 (2)	C18—O3	1.4264 (16)
C7—H7	0.93	C18—H18A	0.96
C8—C9	1.374 (2)	C18—H18B	0.96
C8—H8	0.93	C18—H18C	0.96
			
O2—C1—C9	116.60 (12)	C3—C10—C11	128.70 (13)
O2—C1—C5	122.87 (13)	C3—C10—H10	115.6
C9—C1—C5	120.44 (14)	C11—C10—H10	115.6
O2—C2—C3	112.97 (13)	C16—C11—C12	119.11 (14)
O2—C2—H2A	109	C16—C11—C10	119.20 (14)
C3—C2—H2A	109	C12—C11—C10	121.68 (13)
O2—C2—H2B	109	C13—C12—C11	119.85 (13)
C3—C2—H2B	109	C13—C12—H12	120.1
H2A—C2—H2B	107.8	C11—C12—H12	120.1
C10—C3—C4	119.08 (13)	O3—C13—C12	124.26 (13)
C10—C3—C2	125.45 (14)	O3—C13—C14	115.26 (13)
C4—C3—C2	115.46 (13)	C12—C13—C14	120.48 (14)
O1—C4—C5	122.03 (14)	C15—C14—C13	119.80 (15)
O1—C4—C3	121.80 (13)	C15—C14—H14	120.1
C5—C4—C3	116.12 (12)	C13—C14—H14	120.1
C6—C5—C1	118.60 (14)	C14—C15—C16	120.36 (14)
C6—C5—C4	121.17 (13)	C14—C15—H15	119.8
C1—C5—C4	119.92 (13)	C16—C15—H15	119.8
C7—C6—C5	121.05 (14)	C15—C16—C11	120.37 (15)
C7—C6—H6	119.5	C15—C16—H16	119.8
C5—C6—H6	119.5	C11—C16—H16	119.8
C6—C7—C8	119.27 (15)	O3—C18—H18A	109.5
C6—C7—H7	120.4	O3—C18—H18B	109.5
C8—C7—H7	120.4	H18A—C18—H18B	109.5
C9—C8—C7	121.20 (15)	O3—C18—H18C	109.5
C9—C8—H8	119.4	H18A—C18—H18C	109.5
C7—C8—H8	119.4	H18B—C18—H18C	109.5
C8—C9—C1	119.43 (14)	C1—O2—C2	117.43 (10)
C8—C9—H9	120.3	C13—O3—C18	117.11 (11)
C1—C9—H9	120.3		
			
O2—C2—C3—C10	−136.04 (14)	C5—C1—C9—C8	0.3 (2)
O2—C2—C3—C4	42.74 (17)	C4—C3—C10—C11	178.71 (13)
C10—C3—C4—O1	−19.0 (2)	C2—C3—C10—C11	−2.6 (2)
C2—C3—C4—O1	162.15 (13)	C3—C10—C11—C16	143.03 (15)
C10—C3—C4—C5	158.36 (13)	C3—C10—C11—C12	−38.1 (2)
C2—C3—C4—C5	−20.50 (18)	C16—C11—C12—C13	−2.14 (19)
O2—C1—C5—C6	177.14 (12)	C10—C11—C12—C13	179.04 (12)
C9—C1—C5—C6	0.8 (2)	C11—C12—C13—O3	−178.24 (12)
O2—C1—C5—C4	3.5 (2)	C11—C12—C13—C14	1.8 (2)
C9—C1—C5—C4	−172.79 (13)	O3—C13—C14—C15	179.26 (12)
O1—C4—C5—C6	1.2 (2)	C12—C13—C14—C15	−0.8 (2)
C3—C4—C5—C6	−176.16 (12)	C13—C14—C15—C16	0.1 (2)
O1—C4—C5—C1	174.63 (13)	C14—C15—C16—C11	−0.4 (2)
C3—C4—C5—C1	−2.72 (19)	C12—C11—C16—C15	1.5 (2)
C1—C5—C6—C7	−1.5 (2)	C10—C11—C16—C15	−179.69 (13)
C4—C5—C6—C7	172.06 (14)	C9—C1—O2—C2	−163.08 (12)
C5—C6—C7—C8	0.9 (2)	C5—C1—O2—C2	20.46 (18)
C6—C7—C8—C9	0.3 (2)	C3—C2—O2—C1	−43.00 (16)
C7—C8—C9—C1	−0.9 (2)	C12—C13—O3—C18	1.11 (19)
O2—C1—C9—C8	−176.20 (13)	C14—C13—O3—C18	−178.95 (12)

### Hydrogen-bond geometry (Å, º)

**Table:**

*D*—H···*A*	*D*—H	H···*A*	*D*···*A*	*D*—H···*A*
C2—H2*B*···O1^i^	0.97	2.54	3.3808 (19)	145
C18—H18*B*···O3^ii^	0.96	2.50	3.4227 (19)	161

Symmetry codes: (i) −*x*+1, *y*−1/2, −*z*+1/2; (ii) −*x*, *y*−1/2, −*z*+1/2.
